# An Integrative Model of Self-Regulation in Type 2 Diabetes Self-Management: A Systematic Review of Individual and Family-Based Interventions

**DOI:** 10.3390/healthcare13243230

**Published:** 2025-12-10

**Authors:** Fadli Fadli, Nursalam Nursalam, Elly Lilianty Sjattar, Nilawati Uly

**Affiliations:** 1 Nursing Department, Faculty of Nursing, Universitas Airlangga, Surabaya 60115, Indonesia; 2 Faculty of Health, Mega Buana University, Palopo 91923, Indonesia; 3 Nursing Department, Faculty of Nursing, Hasanuddin University, Kota Makassar 90245, Indonesia

**Keywords:** self-regulation, diabetes self-management, self-care, T2DM

## Abstract

**Highlights:**

**What are the main findings?**

- Self-regulation-based interventions effectively enhance self-care behaviors, knowledge, and self-efficacy among patients with type 2 diabetes.
- The integrative model proposed in this systematic review explains the relationship between disease interpretation, coping strategies, and family involvement in diabetes self-management.

**What are the implications of the main findings?**

- The new integrative model can guide healthcare professionals in designing more comprehensive and patient-centered diabetes education programs.
- Strengthening self-regulation and family support is essential to improve glycemic control and quality of life among patients with type 2 diabetes.

**Abstract:**

**Background/Objectives:** Self-care is essential in managing type 2 diabetes (T2DM), yet it remains suboptimal among patients. This systematic review aimed to determine whether self-regulation-based self-management interventions improve glycemic control, self-efficacy, and quality of life among adults with type 2 diabetes mellitus (T2DM), including individual and family-based approaches. **Methods:** Four major databases (Scopus, ScienceDirect, ProQuest, and PubMed) were systematically searched for English-language studies following PRISMA guidelines. Screening was performed using Rayyan, and study quality was assessed with the JBI critical appraisal tool. Data were synthesized based on PICO outcomes and study design to identify key patterns. The review was registered in PROSPERO (CRD42024594398). **Results:** A total of 881 articles were identified, and 31 met the inclusion criteria. Most studies were randomized controlled trials (54.8%), with diabetes self-management education (DSME) being the most common intervention (41.9%), followed by self-regulation training (12.9%). Nearly half of the studies measured blood glucose and quality-of-life outcomes (22.6%), while others focused on knowledge, behavior, and self-efficacy (19.4%). Only a few studies addressed individual and family-oriented interventions. **Conclusions:** DSME and self-regulation-based approaches are recommended as complementary strategies to improve diabetes self-management. This review introduces a novel integrative model linking disease interpretation, coping strategies, and family support, and highlights their influence on patient self-care behaviors. Future research should empirically test this model to clarify the dynamic interactions among its domains and their effects on glycemic control and health outcomes.

## 1. Introduction

Self-care is a deliberate practice of actions to improve and maintain physical, mental, and emotional health, helping individuals live well, manage stress, and prevent illness [1]. In type 2 diabetes mellitus (T2DM), self-care is essential for maintaining healthy blood glucose levels and preventing complications [2]. However, inadequate self-care capacity often results in poor outcomes, frequently associated with non-adherence to medication, diet, and lifestyle recommendations [3,4]. A major factor underlying these limitations is ineffective self-regulation, which significantly influences quality of life among individuals with T2DM [5].

Self-regulation plays a central role in motivating individuals to engage in sustainable behavioral changes [6]. The Individual and Family Self-Management Theory (IFSMT) provides a practical framework that emphasizes the combined roles of patients and their families in managing diet, exercise, blood glucose monitoring, and medication adherence [7,8]. Integrating family support strengthens patients’ ability to maintain effective disease management and improves quality of life [9,10]. In this review, self-regulation is defined as the capacity of people with T2DM to consciously monitor, control, and adjust their thoughts, emotions, and behaviors to engage in self-care practices consistently, encompassing cognitive, affective, and behavioral processes essential for treatment adherence [10].

To frame the self-regulation conceptually, we draw on Leventhal’s Self-Regulation Model and Baumeister’s Self-Regulation Theory, which explain how illness perceptions and self-control mechanisms influence self-care behaviors [11,12]. Unlike cognitive–behavioral therapy and other professionally driven interventions, which often rely on repeated facilitation, self-regulation approaches emphasize patient autonomy, enabling individuals to set goals, monitor progress, and self-correct over time, making them particularly suitable for chronic diseases such as T2DM [9,13,14].

Previous research has shown that self-regulation among people living with type 2 diabetes is still relatively low, with only about 46.5% demonstrating adequate levels [15]. According to the Common-Sense Model of Illness, self-regulation is shaped by individuals’ cognitive representations of their condition, including beliefs about symptoms, consequences, controllability, and treatment effectiveness. Therefore, the low self-regulation levels observed in this population may reflect maladaptive or inaccurate illness representations, which need to be addressed when designing interventions [16]. Similarly, a study in China reported that adherence to self-care management, including diet, physical activity, health monitoring, and foot care, was mostly in the moderate (50.4%) and low (33.6%) categories [17].

Globally, T2DM accounts for over 90% of all diabetes cases, with 536.6 million individuals affected in 2021 and an estimated 783.2 million by 2045 [18,19]. Prevalence is higher in lower socioeconomic groups and increases with age, particularly affecting those aged 45 and older [20]. These trends underscore the importance of effective self-management interventions that integrate self-regulation principles.

Self-regulation-based interventions grounded in IFSMT have demonstrated improvements in blood glucose levels and quality of life, largely due to the incorporation of family support into daily self-care routines [10]. Emotional regulation therapies, including Acceptance and Commitment Therapy (ACT) and Dialectical Behavior Therapy (DBT), complement these approaches by enhancing mindfulness, acceptance, emotional awareness, and adaptive coping, directly supporting patients in stress management, motivation, and adherence to self-care behaviors [21,22,23]. Techniques such as goal-setting, self-monitoring, and problem-solving further operationalize self-regulation in practical diabetes management [9,24].

Integrating IFSMT-based strategies with emotional regulation approaches provides a holistic method for diabetes self-management, linking theoretical models of self-regulation with practical interventions. By fostering patients’ autonomy and engagement, these approaches translate cognitive and emotional regulation into consistent self-care behaviors, ultimately strengthening long-term disease management.

In light of these findings, this systematic review seeks to provide empirical insights into self-regulation interventions grounded in individual- and family-centered self-management theories as a promising strategy to enhance self-care and quality of life among individuals with T2DM. Furthermore, it proposes a comprehensive conceptual model offering an in-depth perspective on self-regulation mechanisms in T2DM management.

## 2. Materials and Methods

### 2.1. Eligibility Criteria

This systematic review followed the Preferred Reporting Items for Systematic Reviews and Meta-Analyses (PRISMA) guidelines [25] and was registered in PROSPERO (CRD42024594398). The inclusion and exclusion criteria were determined using the PICOS framework as follows:

Inclusion Criteria:

- Population (P): Adults diagnosed with type 2 diabetes mellitus (T2DM).
- Intervention (I): Interventions explicitly designed to enhance self-regulation in the management of type 2 diabetes. These included behavioral, psychosocial, or educational programs that integrated core self-regulation components such as goal-setting, self-monitoring, problem-solving, coping strategies, and appraisal of outcomes [9,26]. Educational programs that provided only didactic information or knowledge transfer, without these self-regulation components, were excluded.
- Comparison (C): Control groups receiving either standard care or non-self-regulation-based interventions.
- Outcomes (O): Quantitative assessment of diabetes self-care behaviors (e.g., knowledge, diet, physical activity, medication adherence, blood glucose levels monitoring, stress management, and foot care), and quality of life measured via validated questionnaires (covering physical, psychological, social, and environmental domains, including FBG and HbA1c levels).
- Study Design (S): Randomized Controlled Trials (RCTs) and quasi-experimental studies.
- Language and Timeframe: Articles published in English between 2014 and 2023.

Exclusion Criteria:

- Non-primary research such as reviews, editorials, conference abstracts, and study protocols,
- Case reports or applied development studies,
- Qualitative studies or studies with purely descriptive designs.

### 2.2. Search Strategy

The literature search for this review was conducted between May and July 2024, and eligible studies were restricted to those published between 2014 and 2023. The search covered four primary electronic databases: Scopus, Science Direct, PubMed, and ProQuest, as recommended for systematic reviews to ensure comprehensiveness. A search of all databases was conducted using the following keywords and Boolean operators: (‘self-regulation’ OR ‘self-regulation model’) AND (individual and family self-management theory’ OR ‘IFSMT’ OR ‘family’) AND (‘self-management’ OR ‘self-care’ OR ‘DSME’) AND (‘type 2 diabetes’ OR ‘DMT2’ OR ‘DM2’). All keywords were matched to Medical Subject Headings (MeSH). The complete search strategy for all resources is available in Refs. [9,10,14,24,26,27,28,29,30,31,32,33,34,35,36,37,38,39,40,41,42,43,44,45,46,47,48,49,50,51].

### 2.3. Study Selection

All authors participated in the selection process by screening studies retrieved from academic databases. A total of 881 articles were identified from four major databases and imported into Rayyan (https://rayyan.ai, accessed on 1 May 2024), an intelligent web-based platform specifically designed for systematic reviews. Rayyan was chosen due to its advantages in enabling blinded, independent screening by multiple reviewers, automatic duplicate detection, and efficient tagging of inclusion/exclusion decisions, thereby improving accuracy and reducing selection bias.

After automatic duplicate removal, 364 studies remained for title and abstract screening. Four authors independently reviewed these using predefined inclusion and exclusion criteria based on the PICOS framework. Studies were included if the intervention explicitly incorporated self-regulation elements into diabetes self-management education, rather than education-only programs.

**Figure 1:**
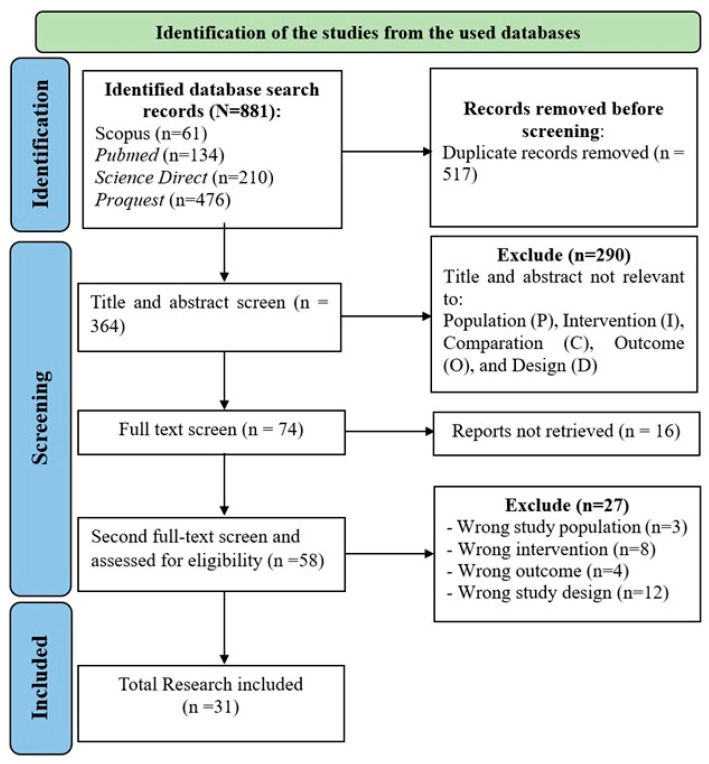
Study Selection (adapted from the PRISMA 2020 Flow Diagram) [25].

A total of 74 articles underwent full-text review. Of these, 58 studies met the eligibility criteria and were further analyzed. Any disagreements among the review team were resolved through discussion and, if necessary, adjudicated by a third, independent reviewer. A consensus level of 80% or higher was considered to indicate strong inter-reviewer agreement [52,53]. Finally, 31 studies were included in the review, as illustrated in the PRISMA flow diagram (Figure 1).

### 2.4. Data Extraction, Analysis, and Synthesis

Data from each eligible study were extracted into a standardized Microsoft Excel spreadsheet. To ensure accuracy, data extraction was independently performed by two reviewers. Extracted items included study characteristics, participant demographics, intervention content, duration, and reported outcomes. Discrepancies were discussed and resolved by consensus, with adjudication by a third reviewer when required.

Due to substantial heterogeneity in the types and durations of self-regulation or self-management interventions, study designs, and reported outcomes, meta-analysis was not feasible. Instead, a narrative and thematic synthesis approach was adopted. Following the PICO framework, the extracted data were first organized into the following categories: Population, Intervention, Comparison, Outcomes, and Study Design. Next, an inductive thematic synthesis was conducted following (1) line-by-line coding of relevant findings, (2) grouping codes into descriptive categories, and (3) generating higher-order analytical themes. Coding was performed independently by two reviewers and then cross-checked for consistency. Final themes were refined and agreed upon through iterative team discussions. This process ensured transparency and minimized bias in synthesizing the evidence base [54].

### 2.5. Risk of Bias and Study Quality

The methodological quality of the included studies was evaluated using the JBI Critical Appraisal Tool, applied according to study design. This assessment examined potential sources of bias related to study design, implementation, and analysis. Each study type had its own set of appraisal questions, which were reviewed individually.

The JBI tool covers key methodological aspects, including clarity of research objectives (Q1), appropriateness of the study design (Q2), adequacy of sampling and participant selection (Q3), rigor of data collection procedures (Q4–Q6), strategies for handling confounding factors (Q7–Q8), validity and reliability of outcome measurements (Q9–Q11), and appropriateness of statistical analysis (Q12–Q13).

Quality scores were converted into percentages and categorized as ≥75% = Good, 50–74% = Enough, and <50% = Poor [55]. Only studies meeting acceptable methodological quality were included in the final synthesis.

## 3. Results

### 3.1. Study Characteristics

This systematic review identified 881 studies across all specified databases, of which only 31 met the inclusion criteria. Table 1 describes the percentage of study characteristics that were obtained.

Based on Table 1, most of the included studies were RCTs (17; 54.8%). The interventions applied to type 2 diabetes patients varied considerably, but in this review, they were grouped into two major categories based on their theoretical foundations. Diabetes Self-Management Education (DSME) interventions (13; 41.9%) primarily emphasized structured education to improve diabetes knowledge, dietary adherence, physical activity, medication use, and blood glucose monitoring. Although several DSME programs incorporated elements of self-regulation, such as goal-setting and self-monitoring, their central framework was educational rather than regulatory. By contrast, self-regulation training programs (4; 12.9%) explicitly adopted self-regulation theory as their conceptual basis, focusing on mechanisms such as problem interpretation, goal-setting, emotion regulation, feedback, and appraisal of coping success. Some of these programs also involved family members in order to strengthen patients’ regulatory capacity through social and emotional support. These family-oriented approaches were classified as self-regulation training because their primary focus was on enhancing regulatory processes within both individual and family contexts.

Although DSME was the most frequently used intervention, only a small number of studies explicitly used self-regulation as the theoretical model. This overlap illustrates a broader challenge in the literature, where DSME and self-regulation interventions often share components, making clear distinctions difficult. This also highlights an important methodological implication: while DSME emphasizes structured education, incorporating regulatory elements blurs the boundaries between educational and psychological approaches. Furthermore, behavior change interventions often require long-term follow-up and comprehensive psychological assessments, which may not be feasible in all settings [28]. Consequently, the interpretation of findings across studies must take into account the heterogeneity of intervention design and outcome measures, an issue that will be further addressed in Section 4.

### 3.2. Risk of Bias and Study Quality

Of the 31 included studies, 28 (90.3%) were rated “Good” quality, with a JBI critical appraisal score above 75.0%. These studies demonstrated clear methodological rigor, including well-described interventions, valid outcome measurements, and appropriate statistical analysis. However, three studies (9.7%) received a “Moderate” rating due to methodological limitations, such as unclear randomization procedures, small sample sizes, and lack of blinding. Despite these limitations, the studies were included due to their relevance, and their findings were interpreted with caution in the synthesis. The full assessment is presented in Table 2.

### 3.3. Impact of Intervention on Type 2 Diabetes Patients

A review of 18 studies found that diabetes self-management interventions improved knowledge, self-management behaviors, self-efficacy, emotional responses, quality of life, and glycemic control among patients with type 2 diabetes. Furthermore, six studies applied information system-based self-management interventions to improve behavior and quality of life. In another intervention, four studies applied self-regulation training programs to improve knowledge, self-management behaviors, self-efficacy, emotional responses, quality of life, and glycemic control in patients with DM type 2. Meanwhile, three studies included individual and family interventions to improve self-management. This showed the need to develop an intervention that includes one family member in self-management to improve health status.

#### 3.3.1. Thematic Synthesis

An inductive thematic synthesis was conducted to integrate findings from the included studies. The analysis involved line-by-line coding, grouping codes into descriptive categories, and generating higher-order analytical themes. To enhance transparency and reproducibility, we provide a thematic development matrix summarizing the progression from initial codes to categories and final themes (Table 3). This table forms the foundation for interpreting the intervention impacts described in the subsequent sections.

The thematic synthesis generated seven interrelated themes that represent the core mechanisms by which self-management and self-regulation interventions influence outcomes among individuals with type 2 diabetes. Collectively, these themes highlight that cognitive preparation through education, behavior modification strategies, and strengthened self-efficacy form the foundational components of effective self-regulation. Family engagement and multi-modal health system support further enhance intervention uptake and continuity. Notably, metabolic improvements were consistently associated with interventions incorporating goal-setting, self-monitoring, appraisal processes, and emotional coping. Together, these themes underpin the development of the integrative model by illustrating the multilevel interactions between individual, family, and healthcare system factors that shape diabetes self-management (Table 3).

#### 3.3.2. Impact of Diabetes Self-Management Intervention

Nearly all studies in this systematic review evaluated the effectiveness of DSME interventions on knowledge, self-management behaviors, self-efficacy, emotional responses, quality of life, and glycemic control in people with type 2 diabetes. Interventions delivered through 2 h of weekly face-to-face sessions over 3 months demonstrated improvements in self-management behaviors, self-efficacy, and reductions in HbA1c [24,31,32,33,46,50]. Some studies also reported sustained benefits for up to 6 months [27,34,35,41,42,56], while DSME programs implemented for 9–12 months showed continued improvements in quality of life [43,44,47].

However, the use of varying intervention durations and educational structures makes direct comparison across studies challenging. While this variation reflects the flexibility of DSME programs to adapt to different clinical and community settings, it also limits the ability to draw standardized conclusions regarding the optimal “dose” of DSME. The heterogeneity of intervention length and content suggests the need for more consistent reporting and standardized frameworks in future studies to strengthen comparability and synthesis of outcomes. Additionally, several studies have shown that patients who regularly receive DSME tend to experience fewer complications, better glycemic control, and improved quality of life, particularly when structured tools such as the Diabetes Self-Management Questionnaire (DSMQ) are used [57].

This study demonstrates that different questionnaires are used across studies that assess the outcomes of the DSME intervention. Specifically, questions were developed based on the Diabetes Knowledge Scale (DKS) [58]. To determine indicators of self-care management activity through the Diabetes Self-Care Activities Scale (SDSCA) questionnaire [59]. Meanwhile, patients’ emotional responses were measured using the Self-Rating Anxiety Scale (SAS) [60] and the Self-Rating Depression Scale (SDS) [61]. The results show a significant impact on quality of life, as measured by the WHOQOL-BREF [62] and the Diabetes Quality of Life (DQOL) [63]. Therefore, this systematic review demonstrates that DSME intervention outcomes can be measured using several questionnaire modifications.

#### 3.3.3. Impact of the Self-Regulation Training Program Intervention

Based on this systematic review, four studies implemented self-regulation interventions to improve diabetes knowledge, self-management behaviors, self-efficacy, emotional responses, quality of life, and glycemic control among patients with type 2 diabetes [9,10,14,24]. These interventions typically consisted of multiple components, such as educational sessions and behavioral skill-building activities delivered over 3 weeks, and were shown to effectively improve self-management behaviors and reduce HbA1c levels [9,10,14].

There were no statistically significant changes in HbA1c at 12-month follow-up in one study, although clinical improvements were observed. This may be attributable to factors such as environmental influences, small sample size, or the long duration between intervention and evaluation [29]. Other studies showed that family-oriented self-management programs produced favorable effects on HbA1c [26], emphasizing the important role of family in supporting diabetes management. A study from China also reported that such interventions were effective in community health centers for reducing HbA1c after approximately 3 months [36], as summarized in Table 4.

### 3.4. Development of a New Integrative Model

**Figure 2:**
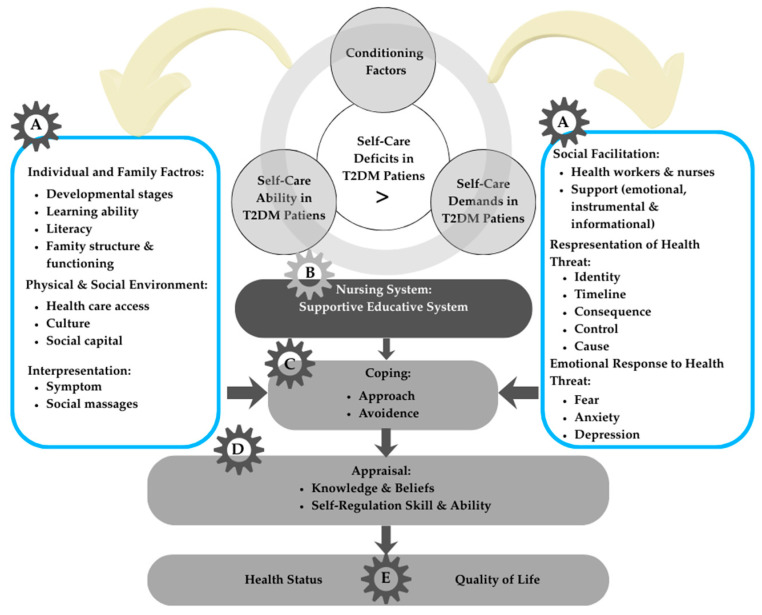
Integrative model of self-regulation in T2DM self-management. Letters denote model components: A—Conditioning factors; B—Interaction between self-care ability and demands; C—Coping strategies (approach or avoidance); D—Appraisal process (knowledge, beliefs, and self-regulation skills); E—Outcomes (health status, quality of life, and cost of health).

Although this review demonstrated that both Diabetes Self-Management Education (DSME) and self-regulation training interventions positively influenced knowledge, self-care behaviors, self-efficacy, quality of life, and glycemic control, it also revealed substantial overlap between the two approaches. Many DSME programs included self-regulation components such as goal-setting and self-monitoring, while self-regulation training interventions sometimes incorporated educational aspects. This overlap highlighted the difficulty of drawing a clear distinction between DSME and self-regulation programs and pointed to the need for an integrative framework that captures their shared mechanisms while accounting for contextual influences from individual, family, and environmental factors. To address this gap, a new integrative model was developed that synthesizes elements of self-regulation theory, the IFSMT, and self-care frameworks, as presented in Figure 2.

The factors discussed below describe a new integrative model that explains the relationships among the self-regulation model, individual and family self-management theory (IFSMT), and self-care interventions in patients with type 2 diabetes. The development of this new integrative model is structured into five domains, derived from the systematic review and the synthesis of multiple theoretical frameworks, as illustrated in Figure 2.

The integrative model was developed by systematically mapping the seven analytical themes derived from the thematic synthesis (Table 3) onto each model component (Figure 2). This explicit linkage ensures that the framework is empirically grounded, theoretically coherent, and transparently derived from the included studies.

#### 3.4.1. Factors That Influence Individuals and Families with Type 2 Diabetes (Part A)

Changes in self-management behavior are essential for improving the quality of life and preventing complications in individuals with T2DM. According to the IFSMT framework, behavior change requires a contextual foundation comprising risk factors and supportive conditions. In the integrative model, Part A represents conditioning factors that influence a person’s ability to engage in effective self-care. Personal characteristics such as age, gender, socioeconomic status, family history of diabetes, disease severity, and duration shape coping tendencies, as demonstrated in previous studies showing that older age and longer disease duration are associated with lower adherence to self-care [20].

Disease interpretation also arises within this component, involving symptom recognition, understanding treatment needs, and beliefs about disease causes, which have been linked to self-management outcomes [20]. Environmental factors, including physical and social surroundings, influence perceptions and self-management capabilities, with studies highlighting the role of community support and access to resources in facilitating effective diabetes care [64]. Family-related factors, such as knowledge, caregiving skills, family functioning, and health literacy, are key contextual influences determining how individuals and families respond to diabetes challenges, as reported in [57].

#### 3.4.2. Nursing System (Intervention)

In the integrative model (Part B), the nursing system serves as the mechanism through which healthcare professionals provide supportive educational interventions when individuals’ self-care abilities are insufficient to meet their self-care demands [58]. Within the included studies, this system was most commonly operationalized through Diabetes Self-Management Education (DSME), structured self-care programs, and self-regulation–oriented interventions delivered by nurses or multidisciplinary teams [9,14]. While these interventions collectively aim to strengthen self-care ability, their modes of delivery vary, reflecting different emphases on education, behavioral skill-building, emotional regulation, and coping guidance.

Rather than functioning as isolated components, these interventions interacted with the conditioning factors identified in the model, such as individual literacy, emotional responses, family support, and perceptions of disease threat by tailoring the structure and content of nursing strategies. For example, interventions that integrated self-regulation training were more responsive to patients’ emotional and cognitive barriers, addressing fear, anxiety, and maladaptive coping patterns while simultaneously promoting problem-solving and goal-setting. In contrast, DSME-focused interventions tended to prioritize knowledge acquisition and behavioral routines, which benefited individuals with adequate literacy but were less effective for those with high emotional distress or limited self-efficacy.

Across the 18 studies, supportive–educative interventions demonstrated consistent benefits in improving diabetes knowledge, self-management behavior, self-efficacy, quality of life, and glycemic outcomes. However, the degree of improvement varied, partly due to differences in intervention intensity, delivery personnel (nurse-led vs. multidisciplinary), and the extent to which emotional regulation or coping strategies were explicitly included. Four studies that incorporated self-regulation training showed particularly meaningful effects on emotional responses and coping ability, suggesting that addressing psychological processes may be a critical mechanism through which nursing systems enhance adherence and sustained self-care.

Collectively, these findings illustrate that the nursing system functions not merely as a provider of information but as an adaptive intervention mechanism that connects individual needs, emotional and cognitive processes, and behavioral demands. Its effectiveness appears strongest when interventions synthesize educational, behavioral, and emotional components rather than addressing them separately, thereby bridging the gap between self-care capacity and self-care demands within the integrative framework.

#### 3.4.3. Coping and Appraisal Skills

In the integrative framework, Part C represents coping strategies through which individuals manage the demands of diabetes, including approach-oriented behaviors such as problem-solving, emotional regulation, and adherence to treatment routines, as well as avoidance-oriented responses [65,66,67]. The thematic synthesis of the included studies showed that coping was strengthened through three recurring patterns: improved cognitive appraisal of illness demands, enhanced emotion-focused coping, and increased problem-focused coping [10]. Interventions such as DSME, structured self-care programs, and self-regulation training contributed to these changes by improving understanding of illness, reducing diabetes-related distress, and supporting goal-setting and action planning [9].

Part D, appraisal, involves evaluating the effectiveness of these coping responses, and several studies demonstrated that better appraisal was associated with higher self-efficacy, improved adherence, and more stable emotional outcomes [24]. Although Leventhal’s Self-Regulation Model presents appraisal as occurring after coping, both IFSMT and SRM conceptualize this process as iterative, with appraisal continually shaping subsequent coping and interpretation [20]. The integration of study findings within this model indicates that enhancements in coping and appraisal are key mechanisms through which interventions facilitate more effective and sustainable diabetes self-management.

#### 3.4.4. Outcome (Health Status and Quality of Life)

Across the included studies, two outcome themes consistently emerged: health status and quality of life. Health status was reported in all five studies and primarily assessed through glycemic indices, including fasting blood glucose levels and HbA1c, with significant improvements observed following DSME, structured self-care programs, or self-regulation training [10,14,24,34,56].

Quality of life was reported in two studies using the WHOQOL-BREF instrument and showed significant enhancement following DSME-based interventions. In addition, emotional well-being and stress reduction were reported as secondary outcomes in two studies [34,56]. Overall, the evidence indicates that interventions grounded in self-regulation principles positively influence clinical outcomes, emotional responses, and quality of life in individuals with type 2 diabetes. The integrative model derived from the reviewed literature is summarized in Table 4.

## 4. Discussion

This systematic review aimed to evaluate the effectiveness of self-management interventions based on the self-regulation model and to develop an integrative framework to guide future research and practice in type 2 diabetes care. Across the included studies, we found consistent evidence that both self–regulation–based approaches and structured self-management programs improve self-care behavior, self-efficacy, emotional responses, and glycemic control. At the same time, our synthesis revealed substantial overlap among the educational, behavioral, and psychological components embedded in these interventions, highlighting the need for a unified model that captures how individual, family, and systemic factors interact in the self-regulation process. The following sections interpret these findings in relation to existing literature, compare the effectiveness of intervention types, and describe how the integrative model was formulated based on the recurring constructs identified in the review.

### 4.1. Effectiveness of DSME Intervention

This systematic review aimed to evaluate the effectiveness of self-management interventions grounded in self-regulation principles and to map their shared mechanisms, to inform an integrative framework for future diabetes care. Overall, the evidence consistently demonstrates that DSME-based and self-regulation-based interventions improve knowledge, self-care behaviors, self-efficacy, quality of life, and glycemic outcomes in individuals with type 2 diabetes. These findings reinforce the central role of self-regulation processes, particularly goal-setting, self-monitoring, and appraisal, in shaping long-term diabetes self-management.

Diabetes Self-Management Education (DSME) emerged as a structured, evidence-based strategy that facilitates not only knowledge acquisition but also the development of skills and abilities required for sustained self-care [47]. By incorporating key self-regulation components such as goal-setting, self-monitoring, and behavioral feedback, DSME enables individuals to adjust lifestyle practices, enhance motivation, and strengthen self-awareness [9,10,68]. Thus, DSME functions not merely as an educational intervention but as a self-regulation framework that supports continued behavioral optimization.

Across the included studies, DSME interventions produced short-, medium-, and long-term positive effects on self-care knowledge and behavior [26,30,45,47,49]. Programs based on the Orem Self-Care Model and Health Belief Model also contributed to improved self-efficacy and quality of life [38,39,50]. A notable pattern was the rise of technology-assisted DSME, including video-based education, SMS-based reminders, and eHealth family education, which inherently support self-regulation through continuous goal tracking, monitoring, and feedback [31,32,35,36,37,40]. These findings are aligned with recent reviews showing that mHealth interventions facilitate sustained self-management and may serve as cost-effective behavioral tools for chronic disease management [69,70].

Although these findings demonstrate consistent improvements across DSME, SMS-based, mHealth, and family-oriented programs, the comparative pattern suggests that technology-assisted DSME tends to produce faster behavioral gains due to its higher frequency of monitoring and feedback, whereas family-oriented DSME appears more effective for emotional support and long-term adherence. Methodological variability across studies also helps explain differences in outcomes; for instance, mHealth and SMS-based interventions were often delivered with higher intensity and shorter follow-up periods, whereas family-based and in-person DSME programs typically involved longer durations and nurse-led or multidisciplinary delivery formats. These methodological differences likely contribute to the variability in glycemic and behavioral outcomes, clarifying why some studies reported stronger effects than others. Furthermore, instead of general descriptors such as “positive effect,” the evidence indicates specific outcome patterns, such as improved self-efficacy, reductions in diabetes-related distress, and measurable improvements in glycemic control, which provide a more precise understanding of the intervention’s impact.

Family-oriented DSME additionally demonstrated effectiveness in improving HbA1c, self-management behavior, and quality of life [33,36,42,44]. This aligns with broader evidence noting the importance of family support in maintaining new health behaviors and decision-making in diabetes care [71]. Importantly, our synthesis identified substantial conceptual overlap between DSME and self-regulation training interventions. Many DSME programs incorporated self-regulation mechanisms (e.g., goal-setting and monitoring), while self-regulation interventions often included educational components. This suggests that rather than being distinct approaches, DSME and self-regulation training are complementary and can be integrated using shared behavior change techniques.

Recent advances also highlight the potential of emotion regulation therapies such as Acceptance and Commitment Therapy (ACT) and Dialectical Behavior Therapy (DBT), which address emotional distress, mindfulness, and adaptive coping among people with type 2 diabetes [21,22,23]. Integrating elements of ACT and DBT into DSME may strengthen patients’ psychological readiness and resilience, supporting adherence to self-care behaviors. This aligns with the growing recognition that both cognitive and affective domains are critical for achieving sustained behavior change in diabetes management.

### 4.2. Effectiveness of Self-Regulation Model Program Intervention

Evidence from this review indicates that interventions explicitly grounded in the self-regulation model remain limited, with only four of the 31 included studies directly examining their effects on diabetes knowledge, self-management behaviors, self-efficacy, emotional responses, quality of life, and glycemic outcomes. However, despite the small number, these studies consistently demonstrate that self-regulation-based programs can improve key self-management outcomes, particularly when delivered through structured training formats. For instance, short-term self-regulation training delivered over 3 weeks was shown to enhance diabetes self-management behaviors and glycemic control significantly [9,24].

A central mechanism underlying these improvements is the patient’s cognitive representation of diabetes, which shapes how individuals interpret symptoms, evaluate threats, and choose coping strategies. Studies included in this review support prior evidence that accurate cognitive representations are associated with better behavioral regulation and self-management performance [68,72]. Self-regulation interventions seek to strengthen these representations by helping individuals understand their condition, anticipate challenges, and adjust behavior accordingly.

Technology-assisted self-regulation programs, particularly those that use mobile health applications, represent an emerging and promising direction. These interventions emphasize autonomy, continuous monitoring, and adaptive feedback, enabling patients to develop the skills and beliefs required for sustained behavior change [73]. Evidence from one study showed substantial reductions in blood glucose levels from 162.3 mg/dL to 128.9 mg/dL following a three-week self-regulation program grounded in self-regulation theory [10], further highlighting the potential of such approaches.

Across studies, the core components of self-regulation, self-monitoring, decision-making, and behavioral adjustment were consistently associated with improved self-care outcomes [74,75,76]. However, findings also indicate that self-regulation models are most effective when combined with health education, family involvement, or supportive guidance, suggesting that cognitive–behavioral processes alone may not be sufficient to produce long-term adherence without contextual or social reinforcement [9,24]. This aligns with evidence from Egypt and other settings showing that health education enhances adherence and strengthens the behavioral foundations required for self-regulation [77].

Overall, self-regulation interventions grounded in individual and family self-management theory show considerable promise for empowering adults with type 2 diabetes to manage their condition more effectively and independently [68,78]. Given the limited number of available studies, future research should prioritize developing and testing integrated self-regulation models that combine cognitive, emotional, behavioral, and family components to optimize diabetes self-care.

### 4.3. New Integrative Model

The integrative model developed in this review synthesizes concepts from both self-management and self-regulation theory to offer a comprehensive framework for improving type 2 diabetes self-care [7,67]. Previous studies consistently show that self-management strategies improve clinical outcomes [79,80,81], while self-regulation theory emphasizes individuals’ capacity to monitor, evaluate, and adjust their behavior to achieve desired goals [68]. Integrating these two perspectives enables a more holistic understanding of how patients make decisions, monitor progress, and sustain long-term behavioral change [82].

Disease perception plays a central role in this process. According to the Common-Sense Model, individuals construct illness representations based on personal knowledge and experiences, shaping their appraisal and coping responses [22,23]. For people living with type 2 diabetes, accurate disease appraisal facilitates more adaptive problem-solving and self-management behavior [83]. The Individual and Family Self-Management Theory further supports this by outlining how behavioral change occurs through three interconnected pathways: the self-management process (knowledge, beliefs, and skills), proximal outcomes (self-care behaviors), and distal outcomes (quality of life) [84]. Evidence also shows that involving family in health education enhances knowledge and understanding of diabetes, treatment adherence, and glycemic outcomes [85,86,87].

To integrate findings from the included studies, we conducted a thematic synthesis that identified five recurring constructs: (1) individual self-efficacy and motivation, (2) family support and involvement, (3) patient knowledge and beliefs, (4) healthcare provider facilitation, and (5) behavior monitoring and feedback. These constructs were consistently associated with improved glycemic control and quality of life. They were subsequently mapped into an integrative model structured around three domains: context (individual, family, and healthcare system), process (knowledge, beliefs, self-monitoring, intention), and outcomes (self-care behavior and glycemic control), as shown in Figure 2. This approach ensures the model remains theoretically grounded while directly reflecting empirical patterns across studies.

Additional evidence supports the role of empowerment through education and clinician involvement as key determinants of improved dietary adherence, physical activity, and self-management performance [88,89,90]. Patients with higher self-efficacy and stronger behavioral regulation capabilities are more likely to adhere to treatment, maintain self-care routines, and achieve optimal glycemic control [91,92]. The integrative model, therefore, emphasizes that self-regulation requires an interplay among personal intention, self-efficacy, knowledge, and supportive social structures.

Despite these promising findings, this review has several limitations. A meta-analysis could not be conducted due to considerable heterogeneity in study designs, intervention modalities, age groups, and outcome measurement tools. Although the methodological appraisal using the JBI checklist was rigorous, subjective interpretation remains possible. Future reviews should incorporate post-intervention follow-up assessments to evaluate long-term sustainability better. There is also a need for more homogeneous indicators, such as standardized measures of family function, intervention type, and sample size, to strengthen comparability and validity.

Nevertheless, the current review offers several strengths. It represents one of the earliest evidence-based syntheses evaluating diabetes self-regulation interventions within individual and family contexts. It also proposes a new integrative model that may assist clinicians in designing more practical and contextually appropriate interventions by combining cognitive, behavioral, emotional, and social determinants of self-care.

In practice, this integrative model suggests that DSME programs should explicitly incorporate self-regulation components, goal-setting, self-monitoring, feedback, and coping skills training while actively involving family members to provide both emotional and practical support. Interventions lasting 8–12 weeks may be effective for initiating behavioral change, but long-term reinforcement through follow-up sessions or community support is likely necessary to sustain improvements. Incorporating advances from behavioral science, including mindfulness, acceptance, and adaptive coping strategies (e.g., ACT and DBT), may further strengthen psychological resilience and stress management.

Future research should prioritize the development and testing of structured DSME programs that embed self-regulation mechanisms and consider individual- and family-level factors such as coping responses and appraisal processes. Longitudinal studies are particularly needed to examine dynamic interactions among these variables over time. Clinicians are encouraged to integrate self-monitoring, goal-setting, action planning, and family engagement into routine diabetes counseling, while tailoring interventions to cultural and contextual needs.

Overall, the proposed integrative model advances existing theory by embedding self-regulation within a broader socioecological system. While traditional self-regulation theory concentrates on individual cognitive and behavioral processes, this model extends its scope to include family dynamics, appraisal processes, social and environmental supports, and healthcare system factors. This multidimensional orientation offers a more comprehensive and pragmatically relevant framework to guide the development of future clinical and community-based interventions for type 2 diabetes.

## 5. Implications for Practice

The findings of this review suggest that integrating self-regulation models into diabetes education programs can significantly improve patient autonomy, self-efficacy, and glycemic control. These results have important implications for nursing practice, particularly in community and primary care settings where nurses often serve as the primary providers of diabetes education and behavioral support. Implementing this model in clinical practice requires a coordinated multidisciplinary approach. Nurses and diabetes educators can lead structured DSME sessions and incorporate self-regulation strategies such as goal-setting, self-monitoring, feedback, and action planning.

Self-regulation-based interventions may be most beneficial when introduced early, such as at the time of diagnosis, but can also be integrated into routine follow-up visits or ongoing DSME sessions. Successful implementation requires adequate resources, including trained personnel, culturally appropriate educational materials, and digital tools that support monitoring and behavioral feedback. It is equally important to consider the role of family members, as family involvement was shown to enhance motivation, emotional support, and adherence to self-care routines. Clarifying these practical considerations, who should deliver the intervention, when it should be applied, and what resources are required provides a clearer translational pathway for integrating self-regulation strategies into routine diabetes care and aligns with the reviewer’s recommendation for deeper reflection on implementation within multidisciplinary care teams.

## 6. Conclusions

The findings show that interventions incorporating self-regulation components such as coping strategies, illness appraisal, goal-setting, self-monitoring, and family involvement consistently improve self-care behaviors, glycemic control, and psychosocial outcomes in individuals with type 2 diabetes.

Based on these synthesized findings, this review proposes an integrative model that connects individual, family, and healthcare system factors with the processes of self-regulation and self-management. This model highlights how personal beliefs, knowledge, self-efficacy, emotional regulation, and supportive environments interact to influence behavioral and clinical outcomes.

While the evidence demonstrates promising effectiveness, variations in study design, intervention intensity, and outcome measures limit comparability across studies. Future research should focus on validating the proposed model, standardizing key indicators, and testing comprehensive DSME programs that embed self-regulation mechanisms and account for family and contextual factors. This integrative framework offers a foundation for advancing nursing practice and guiding the development of more tailored and sustainable diabetes self-management interventions.

## Figures and Tables

**Table 1:** Description of Study Characteristics (n = 31).

Component	Characteristics	(n = 31)	Percentage (%)
Study Design	RCT	17	54.8
	Non-RCT	14	45.2
Intervention Type	Self-regulation training	4	12.9
	Diabetes Self-Management Education (DSME)	13	41.9
	Self-Care Education	5	16.1
	SMS Self-Care Education	5	16.1
	Social Support Intervention	3	9.7
	Self-care Model	1	3.2
Results	Self-Care Behavior	4	12.9
	Control blood sugar	1	3.2
	Glycemic Control	4	12.9
	Knowledge, Self-Care Behavior, and Self-Efficacy	6	19.4
	HbA1c Level and Fasting Blood Sugar	5	16.1
	Emotional Intelligence and HbA1c level	3	9.7
	Quality of life	1	3.2
	Blood glucose level, stress, and quality of life	7	22.6

DSME (Diabetes Self-Management Education); HbA1c (hemoglobin A1c).

**Table 2:** Checklist for Randomized Control Trial and Non-Randomized Control Trial from the JBI.

Studies Include Randomized Control Trials (RCT) (n = 17)																
No.	Author	Q1	Q2	Q3	Q4	Q5	Q6	Q7	Q8	Q9	Q10	Q11	Q12	Q13	Total Score	Category
1	Gathu et al., 2018 [29]	Y	Y	Y	Y	Y	Y	Y	Y	Y	Y	Y	Y	Y	13/13	100.0% (Good)
2	Zheng et al., 2019 [30]	Y	Y	Y	Y	Y	Y	Y	Y	Y	Y	Y	Y	Y	13/13	100.0% (Good)
3	Moghadam et al., 2018 [31]	Y	Y	Y	N	Y	Y	Y	N	Y	Y	Y	Y	Y	11/13	84.6% (Good)
4	Abaza and Marschollek, 2017 [32]	N	N	Y	N	Y	N	Y	Y	Y	Y	Y	Y	Y	9/13	69.0% (Enough)
5	Felix et al., 2020 [26]	Y	Y	Y	N	N	Y	Y	Y	Y	Y	Y	N	Y	10/13	76.9% (Good)
6	Garizábalo-Dávila et al., 2021 [33]	Y	Y	Y	Y	Y	Y	Y	Y	Y	Y	Y	Y	Y	13/13	100.0% (Good)
7	Azami et al., 2018 [34]	Y	Y	Y	Y	Y	Y	Y	Y	Y	Y	Y	Y	Y	13/13	100.0% (Good)
8	Boels et al., 2019 [35]	N	Y	N	Y	Y	Y	Y	Y	Y	Y	Y	Y	Y	11/13	84.6% (Good)
9	Feng et al., 2023 [36]	Y	Y	Y	Y	Y	Y	Y	Y	Y	Y	Y	Y	Y	13/13	100.0% (Good)
10	Kusnanto et al., 2019 [37]	Y	Y	N	N	N	N	Y	Y	Y	Y	Y	N	N	7/13	53.8% (Enough)
11	Cheng et al., 2019 [38]	Y	Y	N	N	N	Y	Y	Y	Y	Y	Y	Y	Y	10/13	76.9% (Good)
12	Rondhianto et al., 2018 [39]	Y	Y	N	N	N	N	Y	Y	Y	Y	Y	N	N	7/13	53.8% (Enough)
13	Maslakpak et al., 2017 [40]	Y	Y	Y	Y	Y	Y	Y	Y	Y	Y	Y	Y	Y	13/13	100.0% (Good)
14	Chai et al., 2018 [41]	Y	Y	Y	Y	Y	Y	Y	Y	Y	Y	Y	Y	Y	13/13	100.0% (Good)
15	McEwen et al., 2019 [42]	Y	Y	Y	N	Y	Y	Y	Y	N	Y	Y	Y	Y	11/13	84.6% (Good)
16	Dobson et al., 2018 [43]	Y	Y	Y	N	N	N	Y	Y	Y	Y	Y	Y	Y	10/13	76.9% (Good)
17	Wichit et al., 2017 [44]	Y	Y	Y	Y	Y	Y	Y	Y	Y	Y	Y	Y	Y	13/13	100.0% (Good)
**Studies Include Non-Randomized Control Trial (Non-RCT) (n = 14)**																
**No** **.**	**Author**	**Q1**	**Q2**	**Q3**	**Q4**	**Q5**	**Q6**	**Q7**	**Q8**	**Q9**					**Total Score**	**Category**
18	Chuman and Hatamochi, 2021 [9]	Y	Y	Y	Y	Y	Y	Y	Y	Y	-	-	-	-	9/9	100.0% (Good)
19	Iawchud et al., 2023 [10]	Y	Y	Y	Y	Y	Y	Y	Y	Y	-	-	-	-	9/9	100.0% (Good)
20	Hariyono and Romli, 2020 [14]	Y	Y	Y	Y	Y	N	Y	Y	Y	-	-	-	-	8/9	88.8% (Good)
21	Hailu et al., 2019 [45]	Y	Y	Y	Y	Y	Y	Y	Y	Y	-	-	-	-	9/9	100.0% (Good)
22	Rusdiana et al., 2018 [46]	Y	Y	Y	N	Y	N	Y	Y	Y	-	-	-	-	7/9	77.8% (Good)
23	Tavakolizadeh et al., 2014 [24]	Y	Y	Y	Y	Y	N	Y	Y	Y	-	-	-	-	8/9	88.8% (Good)
24	Zupa et al., 2021 [47]	Y	Y	Y	Y	Y	Y	Y	Y	Y	-	-	-	-	9/9	100.0% (Good)
25	Rasoul et al., 2019 [27]	Y	Y	Y	Y	Y	Y	Y	Y	Y	-	-	-	-	9/9	100.0% (Good)
26	Lee et al., 2019 [28]	Y	Y	Y	Y	Y	Y	Y	Y	Y	-	-	-	-	9/9	100.0% (Good)
27	Nooseisai et al., 2021 [56]	Y	Y	Y	Y	Y	Y	Y	Y	Y	-	-	-	-	8/9	88.8% (Good)
28	Pamungkas et al., 2020 [48]	Y	Y	Y	Y	Y	Y	Y	Y	Y	-	-	-	-	9/9	100% (Good)
29	Hailu et al., 2019 [45]	Y	Y	Y	Y	Y	Y	Y	Y	Y	-	-	-	-	9/9	100.0% (Good)
30	Ghisi et al., 2020 [49]	Y	Y	Y	Y	Y	N	Y	Y	Y	-	-	-	-	8/9	88.8% (Good)
31	Borji et al., 2017 [50]	Y	Y	Y	Y	Y	Y	Y	Y	Y	-	-	-	-	9/9	100.0% (Good)

Present (Y); Not Applicable (NA); Not Present (N); Randomized Control Trial (RCT); Non-Randomized Control Trial (Non RCT); The Joanna Briggs Institute (JBI).

**Table 3:** Thematic Synthesis.

Initial Codes	Categories	Themes
Increased diabetes knowledge; improved illness understanding	Knowledge acquisition	Theme 1: Education strengthens cognitive foundations for self-regulation
Improved diet, exercise, medication adherence, and self-monitoring	Behavioral change	Theme 2: Structured self-management strategies enhance behavior modification
Increased confidence, motivation, and perceived control	Self-efficacy	Theme 3: Self-efficacy acts as a central mediator in self-regulation processes
Family reminders, shared diet preparation, and emotional support	Family support	Theme 4: Family engagement facilitates sustained self-management
Nurse-led education; digital tools (apps, SMS); video-based modules	Health system facilitation	Theme 5: Multi-modal delivery improves accessibility and continuity
Reduced HbA1c; improved glycemic control	Clinical outcomes and quality of life	Theme 6: Self-regulation interventions yield measurable metabolic improvements
Coping strategies, reduced distress, enhanced emotional regulation	Coping and emotional processes	Theme 7: Emotional adaptation supports long-term maintenance of self-care behaviors

**Table 4:** Summary of Study Descriptions (n = 31).

No.	Author, Year	Country	Design	Sample Size (n) Age Mean ± SD (Min − Max)		Participants and Setting	Intervention Type		Instrument	Outcome	Result
				Intervention Group	Control Group		Intervention Group	Control Group			
1	Chuman and Hatamochi, 2021 [9]	Japan	Quasi-Experiment	n = 19 age = 59.8 ± 6.14	n = 10 age = 64.3 ± 3.95	29 people living with type 2 diabetes were divided into two groups	Self-regulation training was delivered over 3 weeks, consisting of: 6 structured sessions (2 sessions per week); 60–90 min per session; Session components included (diabetes education, cognitive behavioral self-monitoring, and behavioral practice); no formal follow-up was reported	Standard care program for people living with type 2 diabetes	Diabetes self-management behavior scale; Cognitive Behavioral Self-Monitoring Scale: Chronic disease self-efficacy scale	Knowledge, Self-Care Behavior, and Self-Efficacy	Self-regulation training improved knowledge and self-management behaviors compared to the control group.
2	Iawchud et al., 2023 [10]	Thailand	Quasi-Experiment	n = 30 age = 59.5 ± 7.29	n = 30 age = 58.5 ± 6.91	Thirty people living with type 2 diabetes were divided into two groups with ages 35–65 years and fasting blood glucose values 126–182 mg/dL.	Self-regulation theory–based program delivered over 3 weeks, consisting of structured face-to-face educational and behavioral practice sessions.	Standard care program for patients	Diabetes self-management behavior scale	Diabetes self-management; Blood glucose control	The intervention enhanced diabetes self-management behaviors and improved glycemic control.
3	Hariyono and Romli, 2020 [14]	Indonesia	Quasi-Experiment	n = 30 age = 20–55 years	There is no control group	30 people living with type 2 diabetes, ages 35–65 years	Self-regulation treatment was conducted over 3 weeks, consisting of 1 session per week, each lasting 90 min. The program included diabetes education, guidance on self-monitoring, behavioral regulation strategies, and goal-setting activities to improve glycemic control.	No control group	Glycemic Control in Type 2 Diabetes Patients	Control blood sugar	The self-regulation program improved glycemic control in patients with type 2 diabetes.
4	Hailu et al., 2019 [45]	Ethiopia	Quasi-Experiment	n = 116 age = 35–75 years	n = 104 age = 35–75 years	220 type 2 diabetes patients meet the criteria with age > 30 years who use insulin therapy	Diabetes Self-Management Education (DSME) is delivered over 3 weeks, consisting of 3 structured sessions (one per week), each lasting 60 min. The DSME program covered diabetes knowledge, diet management, physical activity, blood glucose monitoring, and foot care. Each session included follow-up reinforcement to support participants’ self-care behaviors.	Standard care program for type 2 diabetes patients	DKS, Summary of Diabetes Self-Care Activity (SDSCA), Stanford Self-Management Resource Center (SMRC)	Diabetes Knowledge, Self-Care Behavior, Diabetes Self-Efficacy	The DSME intervention improved knowledge and several self-care behaviors (diet, exercise, glucose monitoring, and foot care), although it did not significantly improve self-efficacy.
5	Gathu et al., 2018 [29]	Kenya	Randomized Controlled Trial (RCT)	n = 55 age = 50.2 ± 9.93	n = 41 age = 47.5 ± 9.54	96 people living with type 2 diabetes who meet the criteria with age 16–65 years and have HbA1c ≥ 8%	DSME delivered over 6 months, consisting of three 1 h sessions held every six weeks. Sessions included diabetes education, review and reinforcement of key messages, and distribution of self-care materials. Follow-up support was provided through telephone reminders and access to a hotline for consultation.	Following the standard care program in people living with type 2 diabetes	HbA1c from laboratory results; Blood pressure was measured using a calibrated digital tensiometer	HbA1c, blood pressure, body mass index (BMI)	The DSME intervention did not produce significant improvements in glycemic control or other clinical outcomes, suggesting that additional factors such as motivation, attitudes, social support, and self-care behaviors may need to be addressed.
6	Rusdiana et al., 2018 [46]	Indonesia	Quasi-Experiment	n = 80 age = 45–75 years	There is no control group	80 type 2 diabetes patients with age criteria > 40 years	DSME delivered over 8 weeks, consisting of weekly 2 h sessions. The program provided structured diabetes education covering diet, physical activity, blood glucose monitoring, and medication adherence.	There is no control group.	Glycosylated hemoglobin test with Alere Afinion as 100 Analyzer; Fasting blood glucose with a portable measuring device (Gluco DR)	HbA1c and fasting blood glucose	The DSME intervention improved glycemic control, as indicated by reductions in HbA1c and fasting blood glucose levels.
7	Zheng et al., 2019 [30]	China	Randomized Controlled Trial (RCT)	n = 30 age = 52 52 ± 10 46	n = 30 age = 51 92 ± 12.30	Sixty people living with type 2 diabetes who meet the criteria, age > 40, have HbA1c ≥ 65% and fasting blood glucose ≥ 7.0 mmol/L.	DSME delivered over **3** months, consisting of 2 sessions, each lasting 45 min. The program focused on improving self-care behaviors (diet control, physical activity, medication adherence, blood glucose monitoring, and foot care) through structured diabetes education.	Regular education program for 3 months	The SDSCA scale, emotional response of PAID score, and Glycemic control	SDSCA, PAID, blood glucose, and HbA1c	The 2-session DSME program improved self-care behaviors across multiple domains and enhanced glycemic control over 3 months
8	Tavakolizadeh et al., 2014 [24]	Iran	Quasi-Experiment	n = 30 age = 54.53 ± 9.06	n = 30 age = 58.93 ± 11.12	Sixty people living with type 2 diabetes meet the criteria with a minimum of suffering from diabetes type 4 for 4 years and age > 50 years.	Self-regulation training was delivered over 1 month, consisting of 10 sessions, each lasting 65 min. The program included education, behavioral practice, and skill development in self-regulation to improve dietary behavior, physical activity, and glycemic control.	Standard care	The SDSCA scale: Self-regulation questionnaire	Diabetes Self-Management and Blood Glucose	Self-regulation training improved blood sugar control and enhanced key self-care behaviors compared to the control group.
9	Moghadam et al., 2018 [31]	Iran	Randomized Controlled Trial (RCT)	n = 21 age = 48.57 ± 7.89	n = 21 age = 45.42 ± 7.71	Forty-two people living with type 2 diabetes who meet the criteria are between 18 and 60 years old.	Video-based self-care education delivered over eight consecutive weeks, consisting of weekly 90 min sessions. The program focused on diabetes knowledge, glycemic control, stress management, and the enhancement of emotional intelligence.	Following the standard program in the clinic and using leaflets	Bar-On Model of Emotional-Social Intelligence (ESI); Glycemic control	Emotional response and HbA1c	Self-care education improved glycemic control and enhanced emotional intelligence, supporting better stress management in patients with type 2 diabetes.
10	Abaza and Marschollek, 2017 [32]	Egypt	Randomized Controlled Trial (RCT)	n = 34 age = 51.77 ± 9.68	n = 39 age = 51.24 ± 8.66	73 people living with type 2 diabetes who are actively treated at the clinic.	Short Message Service (SMS) based education program for 12 weeks (diet control, physical activity, medication adherence, blood glucose monitoring, and foot care)	Leaflet distribution	Diabetes Self-Care Inventory (SCI); HbA1c levels; blood glucose levels.	Self-Care Behavior, HbA1c levels, and blood glucose,	SMS-based education improved glycemic control and strengthened self-care management among patients with type 2 diabetes
11	Felix et al., 2020 [26]	Egypt	Randomized Controlled Trial (RCT)	n = 94 age = 34.60 ± 13.20	n = 44 age = 33.20 ± 9.30	138 people living with type 2 diabetes and their families.	A culturally tailored DSME program delivered through eight 75 min classes over eight weeks, conducted in participants’ homes. The sessions included didactic diabetes education, food and anatomical models, storytelling, question–and–answer discussions, and culturally specific examples. Data collection occurred at baseline and 9 weeks post-intervention.	Standard education	The SDSCA scale: Glycemic control	Self-care (Diet pattern, physical activity) and A1c	Family-based DSME improved A1c levels and supported healthier dietary and physical activity behaviors, highlighting the essential role of family involvement in diabetes management.
12	Garizábalo-Dávila et al., 2021 [33]	Colombia	Randomized Controlled Trial (RCT)	n = 47 age = 34.60 ± 13.20	n = 47 age = 33.20 ± 9.30	94 people living with type 2 diabetes who are adults and actively receiving treatment at the Barranquilla health center.	Social support–based intervention delivered over 4 weeks, consisting of 40 min sessions. The program focused on enhancing social support to improve diabetes self-management, self-care abilities, and quality of life.	Standard care	The SDSCA scale	Diabetes Self-Management	Social-support–based interventions significantly improved diabetes self-management, enabling patients with type 2 diabetes to enhance their self-care abilities and overall quality of life.
13	Zupa et al., 2021 [47]	United States	Quasi-Experiment	n = 123 age = 62.0 ± 12.0	n = 116 age = 64.0 ± 16.0	239 people living with type 2 diabetes who meet the criteria with age > 50 years and HbA1c values > 8%.	Family-supported self-care education was delivered over 123 days, comprising six structured sessions lasting 90 min each. The program emphasized family involvement to enhance self-efficacy and diabetes self-management. Outcomes were assessed at baseline and 12 months post-intervention.	Standard services	Problem Areas in Diabetes Scale (PAID-5); Adapted Stanford Chronic Disease Self-Efficacy Scale	Knowledge, Self-Care Behavior, and Self-Efficacy	These results showed that patients’ support for intervention programs, including family involvement, can improve self-efficacy and self-management.
14	Azami et al., 2018 [34]	Iran	Randomized Controlled Trial (RCT)	n = 71 age = 55.09 ± 10.16	n = 71 age = 53.49 ± 10.98	142 people living with type 2 diabetes with age > 18 years and no disease complications.	Nurse-assisted DSME delivered over 4 weeks, consisting of four 10 min educational videos (one per week). The videos, produced in Persian, provided general information on diabetes self-management, complication prevention, physical activity and foot care, healthy eating, and healthy living with diabetes. The videos were accompanied by nurse-led coaching and verbal encouragement.	Standard care	DSMQ; WHOQOL-BREF; Centre for Epidemiology Studies Short Depression Scale (CES-D)	Blood glucose level, stress, and quality of life	The nurse-assisted DSME program improved long-term glycemic control and blood glucose levels, while also enhancing emotional responses and quality of life among patients with type 2 diabetes.
15	Rasoul et al., 2019 [27]	Iran	Quasi-Experiment	n = 49 age = 44.63 ± 5.29	n = 49 age = 55.26 ± 4.42	98 type 2 diabetes patients with age > 18 years, who have had diabetes for >5 years, and who can use the web.	Weblog-based self-management education delivered over 20 weeks (5 months). Educational content was posted three times per week, with each educational session lasting 90 min. In addition, the program included 20 exercise-related sessions combining aerobic activity and general physical activity, conducted four times per week for 45 min each.	Following the standard program from the clinic	Diabetes Quality of Life (DQOL)	Quality of life	Web-based self-management education improved quality of life in the intervention group, demonstrating the effectiveness of digital platforms in supporting diabetes self-care.
16	Boels et al., 2019 [35]	Netherlands	Randomized Controlled Trial (RCT)	n = 114 age = 40–70 years	n = 114 age = 40–70 years	228 people living with type 2 diabetes aged 40–70 years, receiving insulin therapy for >3 months and HbA1c > 7%.	SMS-based diabetes education in which participants selected their preferred frequency (2–6 messages per week), educational topics (two or three optional topics in addition to mandatory hypoglycemia content), and program duration (6 or 9 months). The intervention delivered tailored educational messages through a smartphone-based system to support glycemic control and self-management behaviors.	Standard care	Summary of Diabetes Self-Care Activities Measure (SDSCA); Quality of life questionnaire (EQ-5D-5 L)	Blood glucose level and quality of life	SMS-based education delivered over 3–6 months effectively improved blood glucose control and enhanced quality of life, highlighting the value of mobile health tools in supporting diabetes management.
17	Lee et al., 2019 [28]	Korea	Quasi-Experiment	n = 30 age = 53.77 ± 9.22	n = 30 age = 53.60 ± 9.04	60 people living with type 2 diabetes aged 18–70 years, HbA1c > 8%, and suffering from diabetes > 6 months.	Personalized lifestyle education delivered through 60 min sessions, including individualized exercise planning (≥150 min, ≥3 times/week) and dietary regimen guidance. Patients received PM education supported by CGMS results and a diabetes education booklet. A telephone follow-up session was provided two weeks later.	Standard program delivery	Cognitive Behavioral Self-Monitoring Scale; American Association of Diabetes Educators	Self-care behavior, self-efficacy, and HbA1c	Enhanced diabetes-management education improved self-care behaviors, increased self-efficacy, and reduced HbA1c compared with basic diabetes education.
18	Nooseisai et al., 2021 [56]	Thailand	Quasi-Experiment	n = 39 age = 58.9 ± 5.15	n = 38 age = 58.82 ± 4.32	77 female people living with type 2 diabetes aged 50–60 years and HbA1c > 7%.	DSME delivered 3 days per week for 4 months, focusing on diabetes education, stress reduction, and self-care skill development.	Standard care	WHOQOL-BREF; Stress Test-20 (SPST—20)	Blood glucose level, stress, and quality of life	DSME lowered blood glucose levels, reduced stress, and improved quality of life among adult women with type 2 diabetes.
19	Hurst et al., 2020 [51]	Thailand	Quasi-Experiment	n = 367 age = 5.16 ± 10.94	There is no control group	Type 2 diabetes patients who are actively taking medication with an age of >20 years.	DSME delivered over 3 weeks, consisting of two sessions per week, each lasting 60–90 min. The program focused on improving diabetes self-management skills, self-efficacy, glycemic control, and patient knowledge.	There is no control group.	Diabetes self-management (SDSCA), Diabetes management self-efficacy (DMSE), Diabetes knowledge (DK)	Diabetes self-management, self-efficacy, knowledge, and glycemic control	The results showed that the diabetes self-management program intervention improved glycemic control, care management, self-efficacy, and knowledge.
20	Pamungkas et al., 2020 [48]	Indonesia	Quasi-Experiment	n = 30 age = 56.5 ± 7.6	n = 30 age = 54.2 ± 9.20	60 people living with type 2 diabetes aged 35–59 years and HbA1c > 6.5%.	Self-management-based coaching program delivered over 12 weeks, with three sessions per week, each session lasting 60 min, including individual follow-up calls once a week to reinforce learning and monitor progress.	Routine program at the Health Center	Socio-demographic and Health Information (SDHI); DSMQ	Diabetes self-management and HbA1c	Coaching based on diabetes self-management principles improved self-management practices and reduced glycemic levels, demonstrating its feasibility and effectiveness for patients with type 2 diabetes.
21	Feng et al., 2023 [36]	China	Randomized Controlled Trial (RCT)	n = 113 age = 65.7 ± 6.7	n = 112 age = 65.4 ± 7.5	225 type 2 diabetes patients aged 18–79 years, HbA1c > 7%, and living with family	Family-based health education eHealth program delivered over 3 months, with two sessions per week, each session lasting 60 min, including weekly follow-up via messaging or phone calls to reinforce learning and monitor family support.	Standard care	Summary of Diabetes Self-Care Activities Scale; Diabetes Family Behavior Checklist	Diabetes Self-Care Activities, Family Support, and HbA1c	Family-based eHealth interventions improved self-care activities, strengthened family support, and reduced HbA1c levels, indicating suitability for use in community health center services.
22	Kusnanto et al., 2019 [37]	Indonesia	Randomized Controlled Trial (RCT)	n = 15 age = 36–65 years	n = 15 age = 36–65 year	30 people living with type 2 diabetes aged 36–65 years, HbA1c > 7%, have been treated for >3 months, and can operate an Android phone	DSME program based on the diabetes calendar application, 6 times a day for 33-month evaluations	Given leaflet media	Summary of Diabetes Self-Care Activities Measure (SDSCA); Diabetes management self-efficacy scale (DMSES)	Self-efficacy, blood glucose, and HbA1c	The results showed differences between the intervention and control groups in self-efficacy, metabolic control, and lipid control among patients with type 2 diabetes.
23	Ghisi et al., 2020 [49]	Canada	Quasi-Experiment	n = 84 age = 59.8 ± 311.39	There is no control group	84 type 2 diabetes patients with a maximum age of 65 years, who are actively treated at the clinic	24-week DSME program with one supervised class/week (1.5 h: 30 min education + 1 h exercise) and home exercise 6 days/week, delivered by an interdisciplinary team.	There is no Diabetes control group.	Diabetes Education Questionnaire (DATE-Q); Exercise self-efficacy (ESE)	DSME and self-efficacy	The education program increased diabetes management knowledge and enhanced self-efficacy, demonstrating effectiveness in strengthening self-management skills during the 24-week intervention.
24	Cheng et al., 2019 [38]	China	Randomized Controlled Trial (RCT)	n = 121 age = 56.13 ± 10.72	n = 121 age = 53.91 ± 13.01	242 type 2 diabetes patients aged >18 years, HbA1c > 6.5%, can be contacted by landline and have good cognitive	6-week empowerment-based program with one weekly session, including one intake session, two small group discussions, and four phone-based individual consultations, using a written curriculum.	Given standard education only once every 2 weeks	Diabetes Distress Scale (DDS); Diabetes Dependent Quality of Life (ADDQoL) Audit	Emotional response and quality of life	Empowerment-based interventions improved quality of life and reduced diabetes-related stress, indicating their potential for broader, longer-term application across patient age groups.
25	Borji et al., 2017 [50]	Iran	Quasi-Experiment	n = 40 age = 44.30 ± 9.80	n = 40 age = 43.80 ± 11.93	80 type 2 diabetes patients who have been diagnosed for >1 year, with ages 18–65 years.	Education program based on the Orem Self-care Model in 6 sessions, with a duration of 90 min each session and an evaluation time of 12 weeks.	Standard care	QOL survey (SF-36)	Quality of life	The Orem self-care model improved quality of life over 12 weeks, supporting its use as an effective approach to enhance self-care and quality of life in patients with type 2 diabetes.
26	Rondhianto et al., 2018 [39]	Indonesia	Randomized Controlled Trial (RCT)	n = 60 age = 57.50 ± 6.83	n = 60 age = 57.70 ± 5.65	120 type 2 diabetes patients who have been diagnosed >6 months with ages 40–65 years.	Health belief model-based DSME program in 6 sessions, with 120 min each session and 6 weeks of evaluation	Daily care is as usual at the Health Center	Diabetes management self-efficacy scale (DMSES); Diabetes distress scale (DDS); Summary of diabetes self-care activities (SDSCA); Diabetes quality of life scale (DQOL)	Self-efficacy, self-care behavior, and diabetes distress, and quality of life	HBM-based educational interventions improved self-efficacy, strengthened self-care behaviors, enhanced quality of life, and reduced diabetes distress, supporting their use as an effective DSME approach for patients with type 2 diabetes.
27	Maslakpak et al., 2017 [40]	Iran	Randomized Controlled Trial (RCT)	n = 30 age = 49.46 ± 4.76	n = 30 age = 50.60 ± 3.74	60 patients with type 2 diabetes, ages 18–55 years, with no psychiatric disorders.	Family-oriented face-to-face and telephone-based education program twice a week, with 30 min each session and 3 months of evaluation	Standard care	Summary of diabetes self-care activities (SDSCA)	Self-Care Behavior and HbA1c	Family-oriented education delivered through face-to-face and telephone-based programs was highly effective in improving self-care behaviors and enhancing glycemic control among patients with type 2 diabetes.
28	Chai et al., 2018 [41]	China	Randomized Controlled Trial (RCT)	n = 63 age = 55.00 ± 7 0.0	n = 55 age = 53.00 ± 9.00	118 people living with type 2 diabetes with ages > 18 years and no psychological diseases.	DSME program once a week for 120 min and 6 months of evaluation	Standard care with 5–10 min of education	SAS; SDS	Emotional response and blood glucose levels	Self-management education significantly improved psychological status and reduced depression, anxiety, and blood glucose levels.
29	McEwen et al., 2019 [42]	United States	Randomized Controlled Trial (RCT)	n = 83 age = 53.64 ± 9.60	n = 74 age = 53.41 ± 8.40	157 type 2 diabetes patients diagnosed >1 year, with ages 35–74 years, HbA1c 8%, and at least one family member participated	Family-based diabetes intervention once a week for 120 min and 12 weeks of evaluation	Standard care with education every 3 weeks	Family Efficacy for Diabetes Scale (FSE)	Self-Efficacy for Health Behaviors	Family-based intervention improved self-efficacy, particularly physical activity, and strengthened family support in managing type 2 diabetes. Thus, involving family members is essential to enhance diabetes self-management.
30	Dobson et al., 2018 [43]	New Zealand	Randomized Controlled Trial (RCT)	n = 177 age = 47.0 ± 15.0	n = 177 age = 47.0 ± 15.0	354 type 2 diabetes patients with ages > 16 years and HbA1c > 8%	Text message-based diabetes self-management support program with follow-up phone interview at 9 months; HbA1c measured at baseline, 3, 6, and 9 months.	Standard care	Stanford self- Efficacy for diabetes scale (SEDM); summary of diabetes self-care activities (SDSCA; two item diabetes distress scale (DDS2); Health-related quality of life	Self-efficacy, diabetes self-care behaviors, diabetes distress, and quality of life	A text-message–based diabetes self-management program improved self-efficacy, HbA1c, and self-management behaviors, thereby reducing stress and enhancing quality of life in patients with type 2 diabetes.
31	Wichit et al., 2017 [44]	Thailand	Randomized Controlled Trial (RCT)	n = 70 age = 61.3 ± 11.6	n = 70 age = 55.5 ± 10.5	140 people living with type 2 diabetes, ages > 35 years, fasting blood glucose > 140 mg/dL, and living with family	3-group education sessions delivered at baseline, Week 5, and Week 9, 2 h per session (1 h interactive learning + 1 h discussion), for groups of 8–12 patient–family dyads, facilitated by a registered nurse; included a Diabetes Information Workbook.	Standard care	Diabetes Self-Care Activities Scale (SDSCA); Diabetes Management Self-Efficacy Scale (DMSES); Physical Component Summary (PCS)	Self-efficacy, quality of life, and glycemic control	Family-oriented self-management intervention programs improved self-efficacy among patients with type 2 diabetes and reduced HbA1c, thereby improving quality of life. Therefore, in conducting DSME, the family should be included in the self-management of type 2 diabetes patients.

Note: ADDQoL: Audit Diabetes Dependent Quality of Life; BMI: body mass index; CES-D: Centre for Epidemiology Studies Short Depression Scale; DATE-Q: Diabetes Education Questionnaire; DDS: Diabetes Distress Scale; DK: Diabetes knowledge; DKS: Diabetes Knowledge Scale; DSME: Diabetes Self-Management Education; DQOL: Diabetes Quality of Life; ESE: Exercise self-efficacy; ESI: Bar-On Model of Emotional-Social Intelligence; EQ-5D-5L: Quality of life questionnaire; FSE: Family Efficacy for Diabetes Scale; HbA1c: Hemoglobin A1c; PAID-5: Problem Areas in Diabetes Scale; PCS: Physical Component Summary; SDHI: Socio-demographic and Health Information; SCI: Diabetes Self-Care Inventory; SDSCA: Summary of Diabetes Self-Care Activity; SMRC: Stanford Self-Management Resource Center; SPST–20: Stress Test-20; WHOQOL-BREF: Word Health Organization Quality of Life-BREF.

## Data Availability

The data supporting this systematic review are derived from previously published studies and publicly available databases, as cited in the manuscript. No new data were created or analyzed in this study.
